# Methodology of a diabetes prevention translational research project utilizing a community-academic partnership for implementation in an underserved Latino community

**DOI:** 10.1186/1471-2288-9-20

**Published:** 2009-03-13

**Authors:** Philip A Merriam, Trinidad L Tellez, Milagros C Rosal, Barbara C Olendzki, Yunsheng Ma, Sherry L Pagoto, Ira S Ockene

**Affiliations:** 1 Division of Preventive and Behavioral Medicine, Department of Medicine, University of Massachusetts Medical School, Worcester, MA, USA; 2 Greater Lawrence Family Health Center, Lawrence, MA and Department of Family Medicine and Community Health, University of Massachusetts Medical School, Worcester, MA, USA; 3 Division of Cardiovascular Medicine, Department of Medicine, University of Massachusetts Medical School, Worcester, MA, USA

## Abstract

**Background:**

Latinos comprise the largest racial/ethnic group in the United States and have 2–3 times the prevalence of type 2 diabetes mellitus as Caucasians.

**Methods and design:**

The Lawrence Latino Diabetes Prevention Project (LLDPP) is a community-based translational research study which aims to reduce the risk of diabetes among Latinos who have a ≥ 30% probability of developing diabetes in the next 7.5 years per a predictive equation. The project was conducted in Lawrence, Massachusetts, a predominantly Caribbean-origin urban Latino community. Individuals were identified primarily from a community health center's patient panel, screened for study eligibility, randomized to either a usual care or a lifestyle intervention condition, and followed for one year. Like the efficacious Diabetes Prevention Program (DPP), the LLDPP intervention targeted weight loss through dietary change and increased physical activity. However, unlike the DPP, the LLDPP intervention was less intensive, tailored to literacy needs and cultural preferences, and delivered in Spanish. The group format of the intervention (13 group sessions over 1 year) was complemented by 3 individual home visits and was implemented by individuals from the community with training and supervision by a clinical research nutritionist and a behavioral psychologist. Study measures included demographics, Stern predictive equation components (age, gender, ethnicity, fasting glucose, systolic blood pressure, HDL-cholesterol, body mass index, and family history of diabetes), glycosylated hemoglobin, dietary intake, physical activity, depressive symptoms, social support, quality of life, and medication use. Body weight was measured at baseline, 6-months, and one-year; all other measures were assessed at baseline and one-year. All surveys were orally administered in Spanish.

**Results:**

A community-academic partnership enabled the successful recruitment, intervention, and assessment of Latinos at risk of diabetes with a one-year study retention rate of 93%.

**Trial registration:**

NCT00810290

## Background

Latinos are the largest minority group in the United States representing 13.7% of the total population [1]. The Centers for Disease Control analyzed data from the Behavioral Risk Factor Surveillance System (BRFSS) and found that Hispanics continue to have a higher prevalence of diabetes than non-Hispanic whites [2]. Overall, 7.4% of Hispanics in the BRFSS had been told by a doctor that they had diabetes. Given the increasing prevalence of sedentary lifestyle and obesity and their correlation with diabetes and heart disease [3,4], it is likely that the number of individuals with type 2 diabetes mellitus will continue to increase, and that this will be an especially significant burden among Latino communities. Latinos have a very high risk of developing diabetes in their lifetime – a 50% probability for Hispanic women, compared to the approximately 1 in 3 chance for the average American, making primary prevention of type 2 diabetes an important priority in this population [5].

The Diabetes Prevention Program (DPP) was a randomized clinical trial that successfully demonstrated that modest weight loss and increased physical activity could reduce the incidence of diabetes in a group of pre-diabetic patients by 58% [6]. However, both the recruitment methodology and the intervention were very costly. The trial included 27 centers recruiting approximately 1 participant/center/week over a 3 year period at a cost (excluding staff) of approximately $1075 per randomized participant [7]. The DPP intervention began with 16 weekly one-hour individual intervention sessions carried out over 24-weeks and continued with monthly individual and group sessions [7]. The methodology used in the DPP may be too expensive to implement in real world settings, requiring less expensive methods to be developed and tested [8,9].

**Figure 1:**
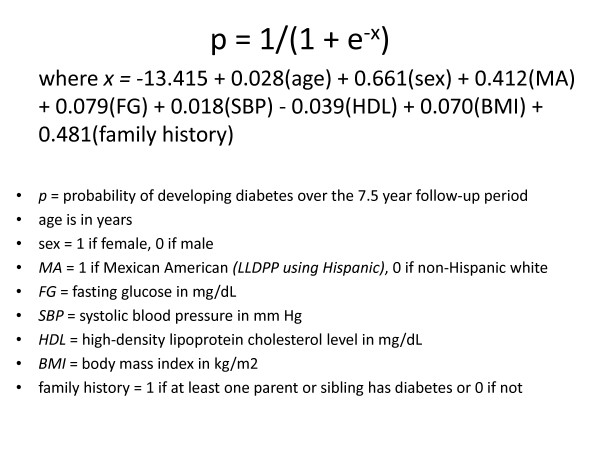
**Stern formula**.

The primary objective of the Lawrence Latino Diabetes Prevention Project (LLDPP) is to design and test a less intensive intervention that, like the DPP, targets weight loss through dietary change and increased physical activity, in order to reduce the risk of type 2 diabetes in a low-income Latino community. The LLDPP study methodology was designed to decrease the high cost of screening and recruitment seen in the DPP, in part by using an accurate but inexpensive screening procedure based on a predictive equation that weights age, gender, ethnicity, fasting blood glucose (FBG), systolic blood pressure, high density lipoprotein (HDL-C), body mass index (BMI), and natal family history of diabetes to estimate relative risk of developing diabetes in the subsequent 7.5 years [10]. See Figure 1 for Stern formula.

The purpose of this paper is to present the methodology used to translate the DPP program to a diabetes-prevention research project in a high-risk Latino community, and to highlight the community-academic partnership formed to carry this out.

## Methods and design

### Setting

This project was carried out in the city of Lawrence, Massachusetts, a primarily Latino community characterized by families struggling with high levels of poverty, limited access to jobs, and limited access to resources families need to prosper [11].

### Community and academic collaborators

Study planning and implementation involved collaboration among the Greater Lawrence Family Health Center (GLFHC), the Lawrence Council on Aging (LCOA)/Senior Center, the YWCA of Greater Lawrence, and investigators from 2 campuses of the University of Massachusetts (UMass). As the study progressed, the Mayor's Health Task Force joined the partnership. The study principal investigator (PI) and a co-PI are UMass Medical School (UMMS) faculty, and the community-PI is a Greater Lawrence Family Health Center (GLFHC) physician and UMMS faculty member. The PI is also a UMass Memorial Medical Center (UMMMC) physician.

The GLFHC provides healthcare to approximately 80% of the Lawrence Latino population. The health center houses a UMMS-affiliated Family Medicine Residency program and a CDC-funded Racial and Ethnic Approaches to Community Health (REACH) diabetes disparities reduction project. The study administrative support for this project was based at GLFHC under the direction of the community-PI, who bridged the partnership among the 6 community and academic collaborators.

**Figure 2:**
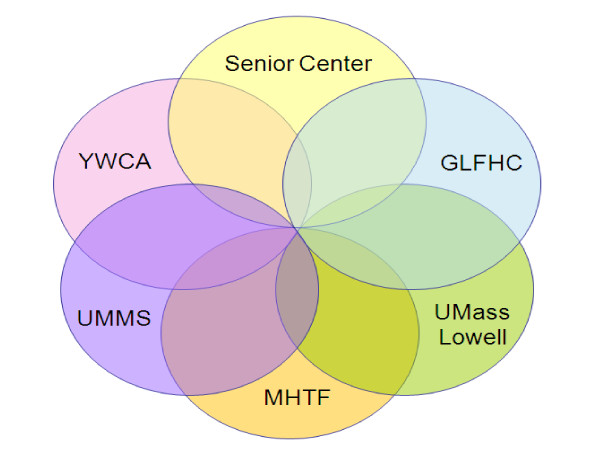
**Study partners**.

The Lawrence Council on Aging (LCOA)/Senior Center, a conveniently located and well-respected social service facility, housed all study screening, recruitment, and assessment appointments. The LCOA and YWCA each provided a community coordinator to staff the study through subcontract arrangements. The community coordinators were chosen for being well-known, respected, and having longstanding relationships and community work experience within the community of Lawrence. Figure 2 presents the study partnership diagram.

### Study management

The partners in this collaboration maintained formal communication through thrice-yearly meetings held at the Lawrence Senior Center, and also informally through regular telephone contacts and e-mail. The study staff was divided into a recruitment/retention team and an intervention team; both teams met monthly. The project director, community-PI, and lead nutritionist shared responsibility for team oversight. The project director, a UMMS faculty member with a background in research management and social work, had primary responsibility for the smooth operation of the study on a day-to-day basis, visiting the clinic at least weekly to meet with study staff, and coordinating the two monthly team meetings. The community-PI, a practicing Latina physician-researcher who was responsible for bridging the community-academic partnership, oversaw the local administrative operations and met regularly with the recruitment/retention team. The lead clinical research nutritionist, who had worked with the co-PI Latina behavioral psychologist to design and develop the intervention materials and train the community intervention team, also met regularly with them [12,13]. Daily management of study activities was facilitated by the use of Lotus Notes/IBM tracking system software (Lotus Notes R5.0.11 ^®^). The database was kept on a server at UMMS which could be accessed easily by study personnel in Lawrence and at UMass. Multiple levels of password protection were used to ensure data security.

### Recruitment and community outreach

The primary recruitment outreach method entailed drawing from the GLFHC patient panel by identifying potentially eligible patients who received a mailed letter of invitation, and then telephone recruitment calls from the study community coordinators. A mailing list was generated every 6 to 8 weeks by running a screening query of the current GLFHC database to identify potentially eligible Latino patients with an age ≥ 25 years who had a high likelihood of meeting eligibility criteria (e.g., overweight, history of hypertension, low HDL-C, or FBG 100–125 mg/dl, and not diabetic). A 2nd query was run to remove patients who had already been approached to be in the study; with approximately 250 names randomly selected for each mailing. Personalized patient screening invitation letters were created, signed by the patient's primary care physician (PCP) and the community-PI, and then mailed. Patients were eliminated by their PCPs if deemed ineligible or a poor study candidate (such as having severe psychiatric illness, etc.). PCPs were kept engaged through regular updates at provider meetings and through communications by the community-PI. The selected GLFHC patient names were downloaded into the Lotus Notes tracking database, and divided equally between the two community coordinators for subsequent telephone outreach.

Additional outreach methods included public service announcements on public access television, guest spots on local Spanish radio programs, advertisements in the local Spanish and bilingual newspapers, flier inserts in the Senior Center newsletter, and mailings to non-GLFHC physicians with the purpose of creating awareness of the study.

### Telephone pre-screening

Pre-screening activities were conducted by each community coordinator who followed up on the mailed invitation letters with telephone calls two weeks after the mailing, unless an individual had already responded and declined further contact. The latter occurred rarely.

The initial telephone contact included an assessment of preliminary eligibility, an invitation to schedule a fasting screening appointment, and if scheduled, instruction to bring in all current medications. Reminder calls were made to patients the day before, and/or the morning of, all scheduled appointments.

### Screening appointment

In addition to those patients scheduled for screening appointments as described above, patients were also screened as walk-ins if they learned of a screening event via word-of-mouth or community outreach.

Following a screening protocol, a community coordinator would explain the study, highlight what would occur at the visit, and obtain a signed screening consent form which was available in English and Spanish. Individuals then were administered a one-page survey which assessed diabetes risk perceptions, and underwent the study screening. All current medications were recorded, and anthropometric measures were taken by the clinic assistant. These included height and weight (without shoes and outerwear), blood pressure (after sitting quietly for 2 minutes) using the Dinamap XL^® ^automated BP monitor, and a fasting fingerstick lipid profile and glucose measure (Cholestech LDX System^®^).

The Stern predictive formula value and BMI were calculated using a Microsoft Excel^® ^program. The community coordinator informed the screened individual of the results of their blood pressure, FBG, weight and BMI, total cholesterol and HDL-C, both verbally and in writing (written into a brief educational brochure), and their potential study eligibility. Patients were informed that their PCP would be receiving the results directly, and were encouraged to review their results with their PCP.

Those Latino individuals who were ≥ 25 years of age, had a BMI ≥ 24 kg/m^2^and a ≥ 30% likelihood of developing diabetes in 7.5 years as predicted by the Stern equation were determined to be pre-eligible and invited to schedule a baseline appointment in 3–4 weeks. Each pre-eligible individual's PCP was mailed a medical clearance form that reviewed the eligibility criteria, and asked for the PCP's permission for the individual to participate.

Through screening or the PCP medical clearance form, the following criteria defined an ineligible state: a fasting glucose of 126 mg/dL or greater, inability or unwillingness to give informed consent, clinically diagnosed diabetes, a plan to move out of the area within the study period, presence of a psychiatric illness which limits ability to participate, no telephone, inability to walk unaided or walk five city blocks (1/4 mile) without stopping, having a medical condition likely to limit lifespan, taking a medication or having a medical condition that interfered with the assessment for diabetes, or having an endocrine disorder that alters blood sugar. In addition to asking these questions on the medical clearance form, all pre-eligible individuals' medication lists and screening flow-sheets were reviewed by the community-PI for study contraindications which included beta-blocking agents (not at stable dose for 3 months or more), thiazide diuretics at doses higher than 25 mg/day, niacin in pharmacologic doses, systemic glucocorticoids, protease inhibitors, atypical antipsychotic agents (not at stable dose for 6 months or more), or prescription weight loss medications.

Nine hundred and forty-nine individuals had screening appointments during the screening phase which spanned 34 months, beginning on October 10, 2004.

### Baseline and follow-up assessments

Recruitment into the study occurred at the baseline appointment. Each individual's community coordinator explained the study again, this time in more detail and highlighting the commitment to three visits over the one-year study period; and a second study consent form was reviewed and signed. The participant was given a copy of the informed consent, a study brochure, a flier reminding them of the 3 pending telephone assessment calls, and the expected dates of their 6-month and 1 year follow-up appointments. Each participant was also given a food portion visual handout for reference during the telephone assessments and community coordinator contact information. All study participants completed interviewer-administered assessments which included demographic questions (age, gender, education, occupation, and household data), as well as social support (the Medical Outcomes Study scale) [14], depressive symptoms (the Center for Epidemiological Studies Depression Scale (CES-D) [15], and quality of life (SF-12) questions [16].

The baseline assessment visit also included anthropometric measures (weight, height and waist circumference); two blood pressure readings (ten minutes apart); and a fasting venous blood sample for lipid, glucose, and HbA1c assays. Serum and plasma aliquots were prepared and the buffy coat layer saved. A serum aliquot was sent to the University of Massachusetts Lowell for the lipid profile and a plasma aliquot for the glucose assay; and a frozen whole blood sample (with EDTA) was sent to the Diabetes Diagnostic Laboratory at the University of Missouri for analysis of HbA1c measures. Extra serum and plasma was saved from those who provided informed consent for additional studies.

Three randomly selected 24-hour dietary and physical activity assessments (24 HR) [17] (NDSR-2007^®^) were conducted by trained bilingual Spanish-speaking registered dietitians not involved in the intervention and blinded to participant's condition, via unannounced telephone interviews (on two weekdays and one weekend day) within the following 2 weeks of the assessment visit. Study participants were asked to refer to a food portion visuals booklet they had received at the baseline assessment to facilitate portion size estimation.

At 6 months post-baseline, a measure of weight was scheduled. At one year, the measures collected at baseline were repeated and included demographics, one-year questionnaire, Stern predictive equation variables, weight, laboratory measures, and 24 HR.

**Figure 3:**
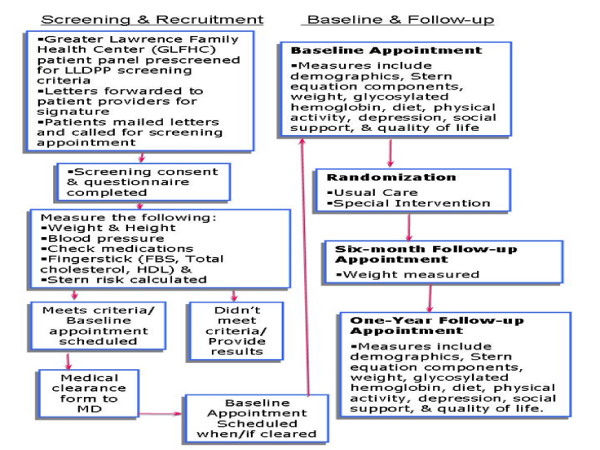
**Screening and assessment process**.

Cash incentives of $25 were given at the baseline visit and the 6-month assessment; and $50 was given for study completion at the one-year assessment. The Institutional Review Boards of the University of Massachusetts Medical School and Greater Lawrence Family Health Center approved the subject recruitment and data collection procedures. The complete process is outlined in Figure 3.

### Randomization and study conditions

After recruitment and completion of baseline data collection, individuals were randomized to receive the usual care (UC) or to a lifestyle intervention (LI) condition. Randomization occurred at the household level. If any new participant was from the same household as someone already in the study, they were assigned to the same condition already assigned to that household.

### Lifestyle intervention

A group-based intervention (13 group sessions) complemented by 3 individual home visits was developed using principles of social learning theory and patient-centered counseling. The intervention was intended to increase awareness of diabetes prevention strategies, foster positive diabetes prevention attitudes (i.e., self-efficacy) and promote healthy lifestyle behaviors in the target Latino population using literacy-sensitive and culturally-tailored strategies and materials. Dietary targets included increasing whole grains and non-starchy vegetables; and reducing sodium, saturated fat, hydrogenated fats, portion sizes, and the refined carbohydrates and starches predominant in the Latino diet. Participants were encouraged to consume several smaller meals throughout the day instead of one or two larger meals, and to decrease caloric density by increasing volume and satiety with vegetables, fiber, fruit, and water intake. The physical activity goal emphasized walking and recommended that participants increase their overall physical activity by 4000 steps/day or to increase physical activity to one hour per day.

The protocol included nutrition education that focused on traditional Latino foods (emphasizing healthy Latino low-fat and high fiber foods) with hands-on opportunities to learn healthy cooking and food shopping skills, goal setting, self-monitoring, problem-solving, and information on opportunities to engage in physical activity. Intervention materials were tailored to low literacy needs and to the primarily Caribbean Latino culture of the study participants. Materials used included a videotape-*novella *(soap-opera format) and discussion guide aimed to enhance attitudes toward diabetes prevention (decreasing ambivalence toward lifestyle change, coping with challenges for behavior change); a colorful "food guide" book which classified pictures of frequently consumed Latino and other foods by the colors of a traffic light (green, yellow and red, classified according to saturated or trans-fat content combined with the glycemic index); low-literacy-tailored goal-setting and self-monitoring worksheets; and food models. All participants were given a pedometer and instructed in its use. Other activities included cooking and consumption of culturally-acceptable meals (at all group sessions), a grocery store tour, and group discussions. Information on safe places for walking and exercise was provided. Significant others (family members or friends living in the participant's household) were invited to attend each group session to elicit home-based support for the implementation of the intervention. Telephone contacts were regularly made to remind participants of group or individual sessions, and to maintain rapport with the participant. At each group session participant goal attainment was reviewed, challenges to adherence were discussed, and solutions were proposed by group participants. Successes by some participants served as models for others. Participants were encouraged to set realistic goals and self-monitor their progress at each session, and were weighed at all sessions. Transportation to sessions was provided as needed.

A manual was developed to guide provider delivery of the sessions. The protocol was implemented by Spanish-speaking community individuals with post high school education and previous training in nutrition. The intervention staff received extensive training in the delivery of the intervention protocol including theoretical background and motivational counseling principles, nutritional and exercise aspects of the intervention, practical strategies to facilitate behavior change, and group management skills. Training involved interactive lectures, role plays and mock sessions aimed at practice of the intervention protocol, and were conducted by the study cardiologist-PI, the behaviorist-co-PI, and the clinical research nutritionist, who also provided ongoing supervision. Booster training sessions were scheduled semiannually.

### Baseline results

Of the 9,959 total telephone screening invitation calls attempted, 2,638 individuals completed a telephone screening call that resulted in 1,296 screening appointments scheduled. The actual number of screening appointments conducted totaled 949 with 391 individuals screening pre-eligible and 312 individuals recruited into the study. The study had proposed recruiting 400 individuals with a 25% dropout rate resulting in a total of 300 expected participants. The study actually recruited 312 individuals with a lower than anticipated drop-out rate of 7% resulting in 290 participants at the end of the one-year study intervention period. All individuals chose to complete the study questionnaire in Spanish.

Baseline characteristics of participants are presented in Table 1. The study population is primarily female (74.4%) with a mean age of 51.9 years old (standard deviation (SD) = 11.3). The average BMI was 34 kg/m^2^, with 36% having a sibling with diabetes (18% of their fathers, 30% of their mothers had diabetes). Fifty-nine percent had less than a high school education, and only 14.6% had attended college. Forty-six percent had CES-D of 16 or greater suggesting clinical depression. Mean systolic blood pressure was 128.7 mmHg, the mean fasting glucose was 105 mg/dl, and the mean HDL-C was 48 mg/dL. Reported average daily caloric intake was 1553 kcal, 57% calories from carbohydrates, 17% from protein, and 27% from fat. Percentage of calories from saturated fat was 8.5% and daily dietary fiber intake was 15 grams. Reported total physical activity expenditure was 28.6 met-hour/day, only 1.1 met-hour/day was from leisure time physical activity. The mean population risk of developing diabetes in the next 7.5 years was 56%, based on the Stern predictive equation.

**Table 1:** Participant baseline characteristics of the Lawrence Latino Diabetes Prevention Project (N = 312)

Demographic variables		N or Mean	% or SD
**Demographic variables**			
Gender (n = 312)			
Male		80	25.6%
Female		232	74.4%
Age (years) (n = 312)		51.9	11.3
Body Mass Index (kg/m^2^) (n = 312)		33.9	5.6
Normal		7	2.2%
Overweight		62	19.9%
Obese		243	77.9%
Education (n = 309)			
Never attended school		6	1.9%
Some elementary school or high school		176	57.0%
High school or GED completed		65	21.0%
Vocational or tech school		17	5.5%
University or College		45	14.6%
Smoking in the past 3 months (n = 306)		35	11.4%
Marital status (n = 307)			
Single		62	20.2%
Married or Living with Partner		159	51.8%
Separated, Divorced or Widowed		86	28.0%
Employment status (n = 311)			
Full-time		102	32.8%
Part-time		41	13.2%
Unemployed		38	12.2%
Disabled		76	24.4%
Retired		23	7.4%
Homemaker		30	9.7%
Volunteer Work		1	0.3%
Family history of diabetes or high blood sugar			
Father (n = 297)			
Yes		52	17.5%
No		221	74.4%
Don't Know		24	8.1%
Mother (n = 297)			
Yes		88	29.6%
No		199	67.0%
Don't Know		10	3.4%
Sibling (n = 297)			
Yes		107	36.0%
No		185	62.3%
Don't Know		5	1.7%
**Psychosocial variable**			
Center for Epidemiological Studies Depression Scale Score (n = 309)			
< 16		167	54.1%
> = 16		142	45.9%
			
**Anthropometric variables**			
Fasting Glucose		104.98	12.11
HDL-C		48.02	10.37
Systolic Blood Pressure		128.7	12.35
Stern index ^a ^(n = 312)		0.56	0.22

	**Mean**	**SD**	

**Dietary intake (n = 299)**			**Recommended** **Values**
Energy intake (kcal/day)	1553.2	584.6	
% calories from carbohydrates	56.6	8.6	45–65%
% calories from protein	17.4	5.0	~15%
% of calories from fat	26.7	6.3	25–30%
% of calories from saturated fat	8.5	2.7	< 7%
Total dietary fiber (g/day)	15.2	7.2	≥ 14 g per 1000 Kcal
			
**Physical activity**			
Total MET†-h/d (n = 306)	28.6	6.3	
Leisure MET-h/d (306)	1.1	1.7	
Occupational MET-h/d (n = 291)	4.6	8.6	
Household MET-h/d (n = 299)	4.4	2.9	

^a ^Stern formula a model using age, sex, ethnicity, fasting glucose level, systolic blood pressure, high density lipoprotein (HDL) cholesterol level, waist, and immediate family history of diabetes to predict 7.5-year incidence of diabetes.

†MET_metabolic equivalent task.

## Discussion

The LLDPP established a community-academic partnership to carry out this T2 translational research project. T2 research struggles with human behavior and organizational inertia, infrastructure and resource constraints, and the messiness of proving the effectiveness of interventions aimed at "moving targets" under conditions that investigators cannot fully control [18]. The DPP intervention has been translated into church [19], weight loss clinic [9], and YWCA [20] settings; however we believe this is the first research study that attempts to implement the DPP intervention into a medically underserved Latino community.

There are a number of differences between the LLDPP and DPP. Along the spectrum of translational research, the DPP was a large-scale efficacy study whereas the LLDPP is a community "effectiveness" intervention study – in an uncontrolled setting and more representative of the real world. The DPP utilized the oral glucose tolerance test as the screening tool, implemented a very intensive one-on-one intervention, was very expensive, and recruited a more educated and English-literate population. The LLDPP uses a less demanding Stern predictive equation as the screening tool; a culturally-adapted, primarily group-based, less-expensive intervention; a less educated study population; and conducts the project entirely in Spanish with all tools designed for oral administration in a population with a high rate of illiteracy in both English and Spanish.

Community-based research presents many unique challenges and requires different research skills to implement and evaluate interventions in real-world settings. A decade ago Israel and colleagues presented a synthesis of key principles of community-based research and explored the major challenges to such research [21]. Principles of practice for academic-practice-research partnerships were also described at that time [22]. More recently, Plowfield and colleagues published critical aspects necessary for effective partnerships between academic institutions and community health agencies that included a commitment up front to time, tactful communication, talented leaders and mutual trust [23].

The LLDPP community-academic partnership took considerable amount of time to establish, and started with the partnership of the GLFHC and UMMS. By housing one of the CDC-sponsored Racial and Ethnic Approaches to Community Health (REACH) projects, the GLFHC had gained experience from the implementation of the REACH 2010 project in Lawrence that was instrumental to the start-up of the LLDPP, and had already developed a good research infrastructure in the Lawrence community which may have enhanced *trust *on the part of the community. As well, *trusted*, *talented *community *leaders *from the REACH 2010 Latino Health Coalition (YWCA and LCOA/Senior Center) were involved in leading the LLDPP community-academic partnership, and were instrumental to the study's success both in planning the recruitment design, and devoting the skilled staff. Recruitment and follow-up went exceptionally well because of the *talent *and dedication of the community coordinators and the clinic assistant, all Lawrence Latinas who enjoyed immediate credibility and *trust *because they were known and familiar in the settings they worked. Additionally, basing the study at the Senior Center gave it a significant presence and visibility in the community. C*ommunication *was maintained through innumerable visits, open communication, and regular, active participation in the clinics at the Senior Center by the project director; a collaborative relationship between the project director and the community-PI; monthly team meetings; and regular (thrice yearly) meetings of the Community Advisory Committee which included all study staff, the community leaders, and the academic investigators, under the leadership of an open, engaged, and community-responsive PI.

The DPP was successfully translated to a Latino community utilizing a community-academic partnership. Although the final outcomes remain to be analyzed, the process of translation appears to work. The LLDPP recruited patients at risk of diabetes using a predictive equation (which saved time and money compared to the oral glucose tolerance test), and carried out the intervention and study assessments completely in Spanish, with a remarkable retention rate of 93%. This was largely due to the community-academic partnership that was formed and the ongoing sensitivity to the needs of the Lawrence Latino community.

## Competing interests

The authors declare that they have no competing interests.

## Authors' contributions

IO, TT, MR, BO and PM participated in conception, and design of the study. IO, PM, TT and BO participated in conducting the study. IO, PM, TT, MR, BO, YM and SP drafted or critically revised the manuscript and IO, PM, TT, MR, BO, YM, and SP read and approved the final manuscript.

## Pre-publication history

The pre-publication history for this paper can be accessed here:

http://www.biomedcentral.com/1471-2288/9/20/prepub
